# Propranolol Administration During Morphine Addiction Attenuates Reinstatement of Drug-Aversive Memories Caused by Exposure to Stressful Stimuli

**DOI:** 10.3390/ph19010033

**Published:** 2025-12-23

**Authors:** Alberto Cánovas-Cabanes, Francisco-Javier Teruel-Fernández, Lucía Fernández-López, Elena Martínez-Laorden, Javier Navarro-Zaragoza, Pilar Almela

**Affiliations:** 1 Department of Pharmacology, CEIR Campus Mare Nostrum (CEIR), University of Murcia, 30120 Murcia, Spain; alberto.canovas2@um.es (A.C.-C.); franciscojavier.teruel@um.es (F.-J.T.-F.); lucia.fernandez2@um.es (L.F.-L.); elena.m.l@um.es (E.M.-L.); palmela@um.es (P.A.); 2 Clinical and Experimental Pharmacology Group, Biomedical Research Institute of Murcia Pascual Parrilla-IMIB, 30120 Murcia, Spain

**Keywords:** propranolol, relapse, addiction, stress

## Abstract

**Background/Objectives:** Situations previously paired with drug use can become conditioned stimuli (i.e., physical stress or psychosocial stress) that elicit intense craving and relapse, even after prolonged abstinence. Previous studies have shown that pharmacological disruption of reconsolidation after memory reactivation could be promising for reducing pathological fear and stress-related responses. For this reason, the aim of this research was to examine the role of β-AR in the retrieval of aversive memories through the potential of β-AR antagonism to mitigate the effects of exposure to stressful stimuli. **Methods:** This question was addressed using a model to assess the re-emergence of an aversive contextual memory induced by both physical stressors (restraint and tail-pinch) and psychosocial stress (social defeat) in morphine- or saline-treated mice previously subjected to a conditioned place aversion (CPA) paradigm, in which naloxone was administered to precipitate opioid withdrawal. To assess the effects of propranolol on aversive memories related to opioid addiction, the number of chamber crossings and the time spent in the naloxone-paired compartment were measured. **Results:** Our results showed that morphine-treated mice spent significantly less time in the naloxone-paired chamber than saline mice during the post-test and after exposure to stressful stimuli, than during the pre-test, showing an effect for aversive memories in addiction. In contrast, when propranolol was administered intraperitoneally 30 min before the exposure to both social and physical stress, the time spent enhanced significantly (*p* < 0.01), supporting a role for propranolol in addiction-related memories. **Conclusions:** These results suggest that propranolol could attenuate the aversive memories that may contribute to relapse to opioid addiction.

## 1. Introduction

Currently, global patterns of opioid consumption remain highly heterogeneous. For instance, high-income countries are responsible for most opioid use, with a median consumption rate of 345.1 morphine milligram equivalents per 1000 inhabitants per day between 2009 and 2019, compared to just 23.6 in other countries and 8.3 in developing countries [1]. In settings such as the United States, high availability has contributed to widespread misuse and addiction, particularly due to synthetic opioids like fentanyl [2]. Indeed, it has been reported in 2022 that synthetic opioids were involved in over two-thirds of the more than 100,000 drug overdose deaths [2].

Alterations in the mechanisms that affect memory are fundamental to both the initiation and maintenance of addiction. In fact, the reinforcing effects of drugs promote the formation of robust associative memories involving key brain regions, including the hippocampus, nucleus accumbens, and amygdala [3]. Therefore, situations and contexts previously paired with drug use can become conditioned stimuli (i.e., physical stress or psychosocial stress) that elicit intense craving and relapse, even after prolonged abstinence. Hence, these stimuli reactivate drug-associated memories and destabilize behavioral control [4].

Memory processes are typically classified according to the stages of acquisition, consolidation, and retrieval [5,6]. A post-retrieval restabilization phase, termed reconsolidation, has also been described [7].

There is another stage named memory retrieval, where reconsolidation happens, contributing to and worsening the persistence of addictive behaviors. During retrieval, memories become transiently labile, providing an opportunity for modification. This phenomenon is particularly relevant for maladaptive, drug-related memories, which may be strengthened or updated during reconsolidation, thus perpetuating addictive behaviors [8]. Pharmacological disruption of reconsolidation after memory reactivation has shown promise for reducing pathological fear and stress-related responses [7,9]. Reconsolidation of drug-associated memories is similarly implicated in relapse in opioid use disorders, as reactivated memories become susceptible to strengthening or alteration, thereby contributing to the enhancement of drug-seeking behavior [10]. Alternatively, various associative learning models, including conditioned place aversion (CPA), can be employed to induce reinstatement by exposing subjects to different stressors [11,12]. CPA facilitates the formation or consolidation of aversive memories by linking the negative emotional effects of withdrawal with the surrounding context.

On the other hand, stimulating or impactful experiences trigger the release of noradrenaline (NA), a neuromodulator that affects cell excitability, synaptic activity, and memory learning and processing [13]. It is well known that noradrenaline binds to receptors α and β. Therefore, previous studies have focused on the role of both receptors in memory, with different researchers proposing a main role for β-adrenergic receptors (β-ARs) in memory formation [14,15]. β-AR antagonism has been shown to prevent the formation of memories associated with fear or stress [16,17], and propranolol administration disrupts recognition memory for novel objects [18]. Conversely, β-ARs activation has been associated with enhanced memory persistence [19]. Regarding the role of these receptors in reconsolidation, findings remain inconsistent, although it has been proposed that this stage could also be avoided through blockade of β-ARs. Several studies report that β-AR antagonism attenuates reconsolidation or facilitates extinction, thereby mitigating relapse in substance use disorders [20,21]. Recently, it has been demonstrated that post-retrieval administration of propranolol produces a persistent deficit in CPP memory retrieval, which subsequently attenuates cocaine-induced reinstatement of drug-seeking behavior [22]. Furthermore, selective blockade of β1-ARs has been shown to disrupt cocaine-associated memory retrieval and retention, underscoring the receptor subtype specificity in modulating these memory processes [23]. Overall, β-ARs may contribute to updating memories by regulating the reconfiguration of recalled memories. Nevertheless, other studies indicate that propranolol does not affect memory reconsolidation [24,25].

Understanding whether stress triggers the retrieval of drug-withdrawal memories through activation of the sympathetic nervous system is important for developing novel therapeutic strategies to prevent relapse. Muscle activity measurements offer a potential readout of such stress responses. In this study, a paradigm to measure the re-emergence of an aversive contextual memory induced by physical (restraint and tail-pinch) and psychosocial (social defeat) stressors was applied in mice previously subjected to the CPA procedure. The aim of this research was to expand knowledge about the role of β-ARs in the retrieval of aversive memories and to assess the potential of β-AR antagonism to mitigate physical and/or psychosocial stress-induced negative memories that contribute to possible relapse.

## 2. Results

### 2.1. Number of Crossings and Time Spent by Chamber During Naloxone-Induced CPA

**Figure 1:**
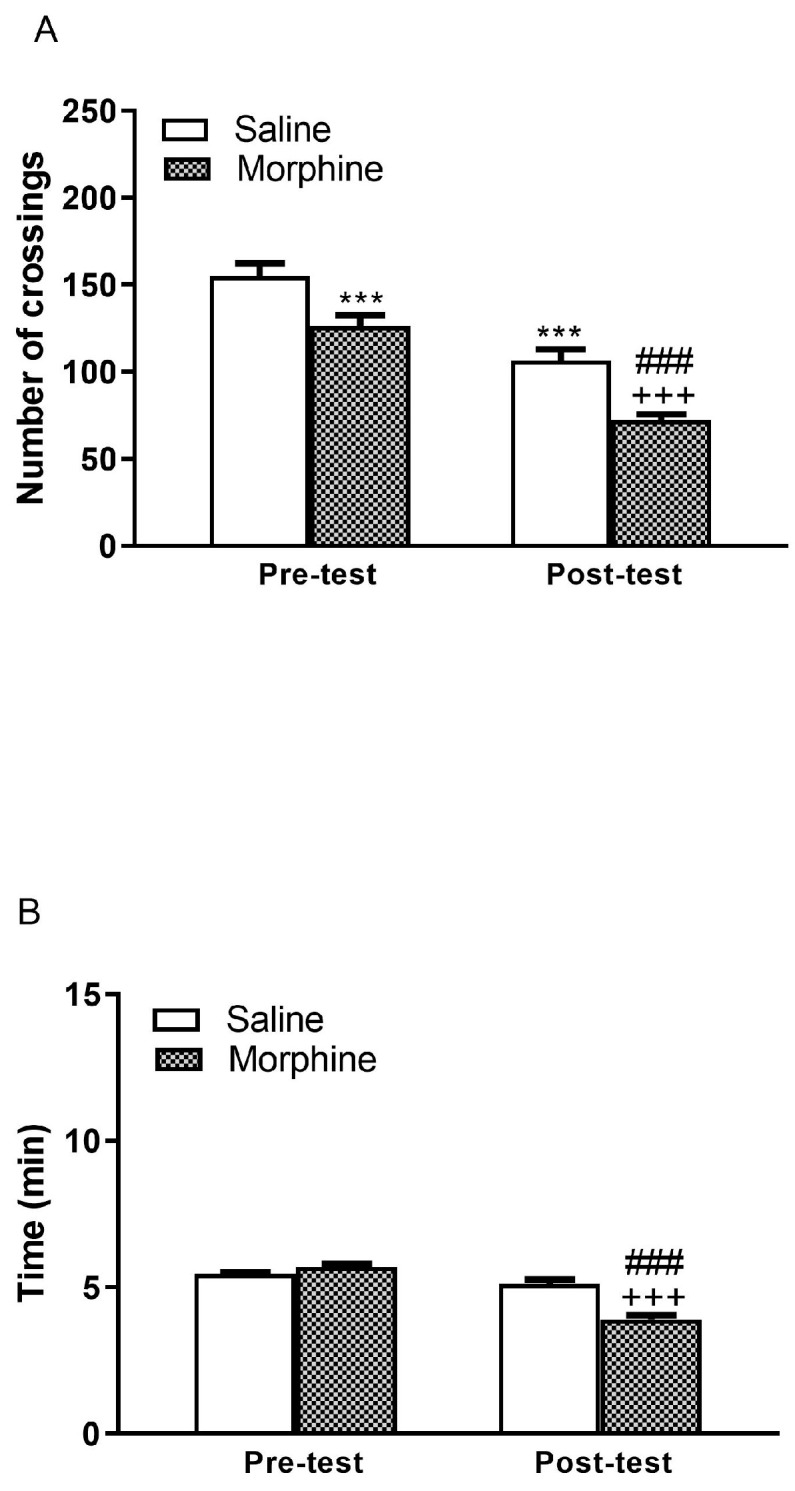
Effects of chronic treatment (morphine or saline) on the number of crossings (**A**) and the time spent by the mice in the naloxone-paired chamber (**B**) during the pre-test and the post-test. Morphine group sample size *n* = 93; Saline group sample size *n* = 86. Administration of naloxone decreased the number of crossings and the time elapsed in the naloxone-paired room. *** *p* < 0.001 vs. saline pre-test, ^+++^ *p* < 0.001 vs. saline post-test, ^###^ *p* < 0.001 vs. morphine pre-test.

The time spent in the randomly assigned chamber (dots or stripes) during the pre-test phase was comparable between the morphine-treated and saline-treated groups (Figure 1B). This indicates that the animals did not show any inherent preference for any chamber prior to conditioning. However, during the test phase, morphine-dependent mice administered naloxone exhibited a significant decrease in crossings compared to the saline-treated control group (*p* < 0.001). Similarly, morphine-dependent mice treated with naloxone spent significantly less time in the naloxone-paired compartments relative to controls (*p* < 0.001) (Figure 1A,B). Mice undergoing morphine withdrawal exhibited characteristic somatic signs, including wet-dog shakes, teeth chattering, piloerection, lacrimation, rhinorrhea, chromodacryorrhea, spontaneous jumping, ptosis, tremor, and diarrhea (Table 1). In addition, saline-treated animals injected with naloxone also showed reductions in total crossings to any compartments (*p* < 0.001) without changes in the duration of stay (Figure 1A).

### 2.2. Number of Crossings and Time Spent by Chamber After Extinction of Naloxone-Induced CPA

**Figure 2:**
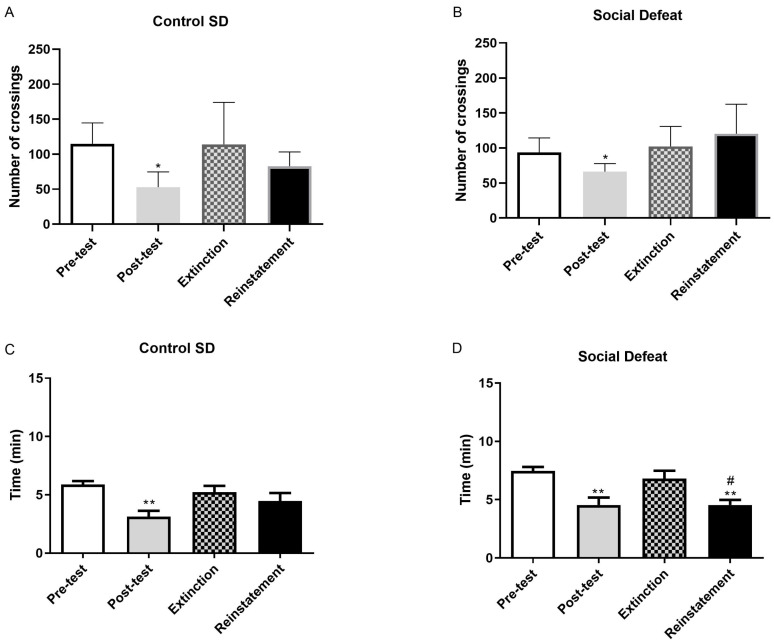
Number of crossings observed during pre-test, post-test, extinction phase, and reinstatement in morphine withdrawal-induced mice (Mor + Nx + Veh) exposed to social stress (**B**) or not (**A**). The post-test phase decreased the number of crossings in both groups. Time spent during these four stages of the CPA test at the naloxone-paired chamber was registered for the animals treated with morphine (Mor) + naloxone (Nx) + vehicle (Veh) and exposed to social defeat (**D**) or the control group (**C**). Naloxone decreased the time spent during the post-test in both groups. Social defeat group sample size *n* = 6; Social defeat control group sample size *n* = 5. Social defeat significantly decreased the time spent in this chamber during reinstatement. * *p* < 0.05 vs. pre-test, ** *p* < 0.01 vs. pre-test, ^#^ *p* < 0.05 vs. extinction.

**Figure 3:**
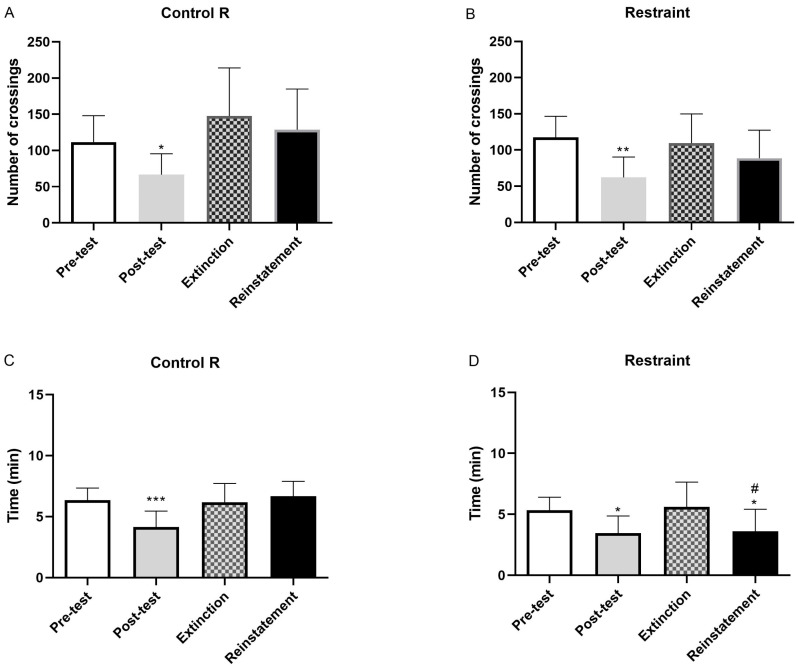
The number of crossings counted during pre-test, post-test, extinction phase, and reinstatement in Mor + Nx + Veh mice exposed to restraint, a type of physical stress (**B**), or not exposed to stress (**A**). The post-test phase showed a decrease in the number of crossings for both groups, regarding the time spent at this chamber when mice suffered restraint (**D**) or absence of stress (**C**). Naloxone decreased the time spent during the post-test in both groups. Restraint group sample size *n* = 9; Restraint control group sample size *n* = 10. Physical stress significantly decreased the time spent in this chamber during reinstatement. * *p* < 0.05 vs. pre-test, ** *p* < 0.01 vs. pre-test, *** *p* < 0.001 vs. pre-test, ^#^ *p* < 0.05 vs. extinction.

**Figure 4:**
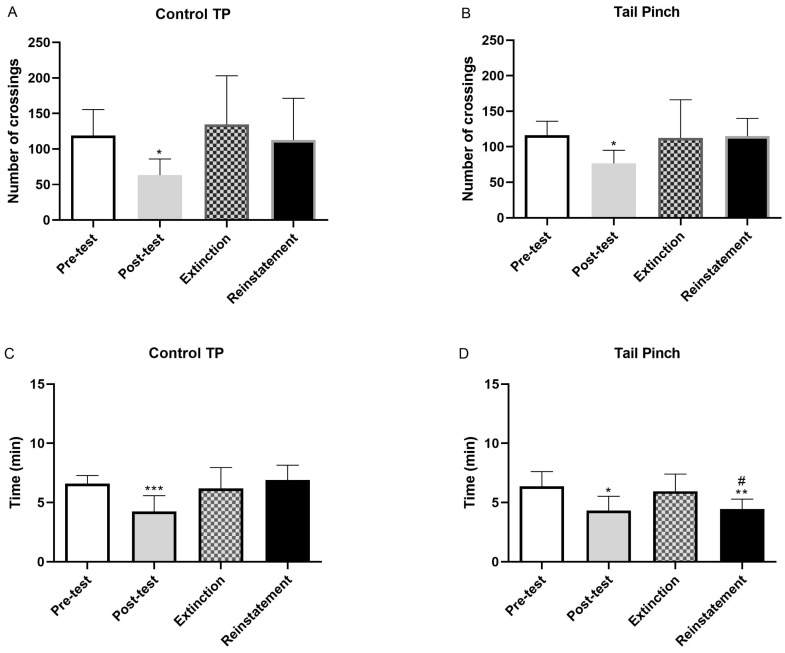
The number of crossings counted during pre-test, post-test, extinction phase, and reinstatement in Mor + Nx + Veh mice exposed to another type of physical stress, tail pinch in this case (**B**), or mice belonging to the control group (**A**). The post-test phase showed a decrease in the number of crossings for both groups. Similarly, the time spent at this chamber when mice suffered tail pinch (**D**) or not (**C**), was decreased during the post-test in both groups. Tail pinch group sample size *n* = 8; Tail pinch control group sample size *n* = 9. Tail pinch significantly decreased the time spent in this chamber during reinstatement. * *p* < 0.05 vs. pre-test, ** *p* < 0.01 vs. pre-test, *** *p* < 0.001 vs. pre-test, ^#^ *p* < 0.05 vs. extinction.

Following the induction of CPA by naloxone, mice underwent consecutive extinction sessions consisting of re-exposure to the conditioning equipment in the absence of naloxone, over the course of the days. The progressive attenuation of naloxone-induced CPA was monitored throughout this period. However, all morphine-dependent animals displayed a significant extinction of aversive behavior over time (*p* < 0.05). The duration and progression of the extinction process did not differ between mice conditioned in either the dotted or striped chambers. No significant differences were observed between groups subsequently exposed or not exposed to stress paradigms (Figure 2, Figure 3 and Figure 4).

### 2.3. Number of Crossings and Time Spent by Chamber After Extinction of Naloxone-Induced CPA and Posterior Exposure to Stressful Stimuli

The present study examined the impact of psychosocial stress—specifically social defeat—and physical stress (restraint or tail pinch) on the context-induced reinstatement of morphine withdrawal-associated memory. To determine whether acute stress could re-establish naloxone-induced conditioned place aversion (CPA), mice were exposed to a single stressor one day after extinction. Figure 2, Figure 3 and Figure 4 depict the reinstatement of naloxone-induced CPA following exposure to distinct stress modalities. As shown in Figure 2, morphine-dependent mice treated with naloxone and vehicle and subsequently exposed to social defeat spent significantly less time in the naloxone-paired compartment during reinstatement compared with the extinction phase (*p* < 0.05). The time spent in the aversive compartment during the reinstatement test was comparable to that observed during the post-test session (Figure 2B). However, the number of crossings did not significantly change following social defeat (Figure 2D). Conversely, no significant differences were observed between extinction and reinstatement in non-stressed animals (Figure 2A,C). Exposure to physical stress produced similar outcomes. Morphine-dependent mice subjected to restraint spent significantly less time in the naloxone-paired compartment during reinstatement relative to extinction (*p* < 0.05). The time spent in the aversive compartment during reinstatement was consistent with the results obtained during the subsequent test (Figure 3B). In contrast, the number of crossings remained unchanged after restraint (Figure 3D). No changes were observed in the non-stressed controls (Figure 3A,C). Likewise, morphine-dependent mice previously conditioned to aversion and exposed to tail pinch spent significantly less time in the naloxone-paired compartment compared with extinction (*p* < 0.05). Time spent in the aversive compartment during the reinstatement test was consistent with the post-test session (Figure 4B). Importantly, the number of crossings was not altered following restraint (Figure 4D), and non-stressed animals again exhibited no significant differences across sessions (Figure 4A,C).

### 2.4. Influence of Propranolol (β-ARs Antagonist) on the Number of Crossings and Time Spent by Chamber After Extinction of Naloxone-Induced CPA and Posterior Exposure to Stressful Stimuli

**Figure 5:**
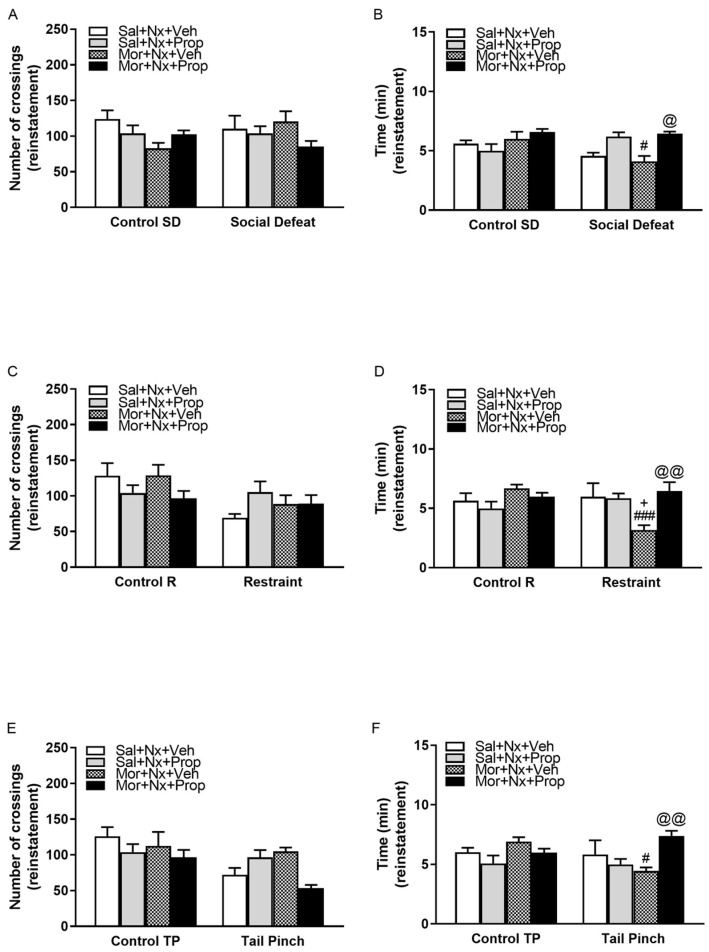
The number of crossings counted during reinstatement in the eight experimental groups when they are exposed or not to social defeat (**A**), restraint (**C**), or tail pinch (**E**). No significant changes were seen for this variable during reinstatement due to the exposure to stress stimuli for any experimental group, even when the mice were previously administered propranolol. In contrast, the time spent at the naloxone-paired chamber when the mice suffered social defeat (**B**), restraint (**D**), or tail pinch (**F**) was significantly increased when the β-adrenergic receptor antagonist propranolol was administered 30 min previously to the exposure to stress, suggesting a blockade of the aversive memories. ^+^ *p* < 0.05 vs. stressed Sal + Nx + Veh, ^#^ *p* < 0.05 vs. non-stressed Mor + Nx + Veh, ^###^ *p* < 0.001 vs. non-stressed Mor + Nx + Veh, ^@^ *p* < 0.05 vs. stressed Mor + Nx + Veh, ^@@^ *p* < 0.01 vs. stressed Mor + Nx + Veh.

As shown in Figure 5 and in Table 2, mice that received saline instead of morphine and were subsequently injected with naloxone showed no significant differences in the CPA reinstatement test after being exposed to psychosocial stress (social defeat), either in the time spent or in the number of crossings. Furthermore, no changes were observed in the saline control group, regardless of stress exposure. Significant effects were detected after administration of the antagonist in the social defeat group. Notably, morphine-dependent mice treated with naloxone and vehicle and subjected to social defeat spent significantly less time (*p* < 0.05) in the chamber paired with naloxone than animals pretreated with propranolol, a β-ARs antagonist, prior to stress exposure (Figure 5B). This indicates that β-AR blockade before stress exposure attenuated the reinstatement of withdrawal-associated memory.

Three-way ANOVA revealed no effects of social stress (*F*(1,45) = 1.143; *p* = 0.2908) or chronic treatment (*F*(1,45) = 0.7451; *p* = 0.3926) by themselves. However, a significant interaction was found for acute treatment (*F*(1,45) = 12.88; *p* = 0.0008. No significant interaction was found between morphine pretreatment and acute treatment (*F*(1,45) = 3.540; *p* = 0.0664). Neither between stress and chronic treatment (*F*(1,45) = 1.400; *p* = 0.2429). However, there was a significant interaction between social defeat and acute treatment (*F*(1,45) = 6.673; *p* = 0.0131). Finally, no significant differences were observed between stress, morphine pretreatment, and acute treatment (*F*(1,45) = 0.6172; *p* = 0.4362). In contrast, the number of crossings was not significantly altered by the injection of propranolol.

Regarding exposure to physical stress stimuli, animals receiving saline and naloxone showed no significant changes in the CPA reinstatement test when exposed to restraint or tail pinch stress. No changes were observed in any of the variables studied (time elapsed, crossings). Similarly, no alterations were observed after the administration of propranolol in these groups (Figure 5C–F).

In morphine-dependent mice subjected to restraint, once the reinstatement test was presented, morphine-dependent mice treated with naloxone and vehicle and exposed to physical stress (restraint) spent significantly less time (*p* < 0.001) in the compartment paired with naloxone, compared to unstressed controls and the saline group (*p* < 0.05) (Figure 5D). However, they spent significantly less time (*p* < 0.01) in the naloxone-paired chamber than animals pretreated with propranolol (Figure 5D), suggesting that β-AR antagonism may prevent stress-induced reinstatement of opioid withdrawal memory. Overall, ANOVA showed no effects of physical stress (restraint) (*F*(1,55) = 1.232; *p* = 0.2718), administration of propranolol (*F*(1,55) = 1.220; *p* = 0.2741 or chronic treatment (*F*(1,55) = 0.0067; *p* = 0.9349). In contrast, ANOVA revealed a significant interaction between restraint and morphine treatment (*F*(1,55) = 6.993; *p* = 0.0106), between restraint and administration of propranolol or vehicle (*F*(1,55 = 7.890; *p* = 0.0069), and between chronic treatment and acute treatment (*F*(1,55) = 4.451; *p* = 0.0394). Moreover, a significant effect was detected between stress, morphine pretreatment, and β-adrenergic antagonist treatment (*F*(1,55) = 4.543; *p* = 0.0375). No significant changes in the number of crossings were seen for any of these groups (Figure 5C).

Finally, morphine-dependent mice treated with naloxone and vehicle and exposed to tail pinch spent significantly less time (*p* < 0.05) in the compartment paired with naloxone compared to the control group (Figure 5F). Consistent with the results for the groups exposed to restraint, morphine-dependent mice treated with naloxone and vehicle and exposed to tail pinch spent significantly less time in the compartment paired with naloxone compared to the group receiving propranolol (*p* < 0.01). This suggests that β-AR blockade reduced both psychosocial and physical stress-induced reinstatement of opioid withdrawal-associated memories.

Three-way ANOVA showed no effects of tail pinch stress exposure (*F*(1,53) = 0.6382; *p* = 0.4279), acute treatment (*F*(1,53) = 0.0184; *p* = 0.8925 or chronic treatment (*F*(1,53) = 2.988; *p* = 0.0897). Regarding the interaction between physical stress and morphine treatment, no significant interaction was observed (*F*(1,53) = 0.2461; *p* = 0.6219). In contrast, significant interactions were found between tail-pinch stress and acute treatment (*F*(1,53) = 5.999; *p* = 0.0177), as well as between chronic and acute treatment (*F*(1,53) = 5.544; *p* = 0.0223). Similarly to the results mentioned above, significant changes were detected among tail-pinch stress, morphine pretreatment, and β-adrenergic antagonist treatment (*F*(1,53) = 5.278; *p* = 0.0256). Finally, no significant changes in the number of crossings between compartments were observed (Figure 5E).

## 3. Discussion

Relapse to drug use after long periods of abstinence remains a significant challenge in the treatment of substance use disorders, and exposure to stressful situations is a major factor contributing to the reinstatement of drug-seeking behavior [26]. The present study aimed to elucidate the impact of psychosocial and physical stress on the reinstatement of morphine withdrawal-associated memory, with a particular focus on the involvement of the noradrenergic system, specifically through the β-ARs. Therefore, it was necessary to ensure the correct establishment of aversion and extinction before the subsequent tests during the reinstatement. Our results confirmed that naloxone induces conditioned place aversion (CPA) in morphine-dependent mice, accompanied by withdrawal signs such as body weight loss, diarrhea, or jumping [27,28]. Administration of naloxone decreased the number of crossings and the time elapsed in the naloxone-paired room (Figure 1). Reduced exploratory behavior and avoidance of the naloxone-paired chamber are consistent with the formation of a robust aversive memory. Furthermore, naloxone administration also elicited CPA in animals that had not received drugs (Figure 1). This aversive response in saline-treated mice may be due to the blockade of tonically released endogenous opioid peptides, a mechanism that appears to depend on stress levels during CPA [29]. We further investigated extinction learning, a mechanism by which memories can be weakened. Consistent with previous studies [30], repeated re-exposure to the compartment paired with naloxone in the absence of the drug led to a reduction in CPA (Figure 2, Figure 3 and Figure 4). Specifically, after several extinction sessions, all morphine-dependent animals treated with naloxone completely extinguished their aversive memory. At the preclinical level, relapse is usually assessed based on measurements of drug-seeking and drug-taking behaviors in experimental reinstatement protocols. These protocols involve the resumption of a previously extinguished drug-reinforced rewarding behavior in response to one or more triggers, such as stress or drug-related cues. Social stressors, in particular, induce a strong motivation to seek the drug, making them potent relapse factors [31]. On the other hand, most existing animal models use physical stressors, such as foot shocks, to resume drug seeking [32]. Taking into account that people with stress disorders are at high risk not only of developing opioid use disorder but also of relapsing after stopping drug use, the way we have displayed to better understand this phenomenon was to conduct CPA tests after a single episode of physical stress (tail pinch or restraint) and psychosocial stress (social defeat) following extinction.

Once the CPA model was considered appropriate, understanding the neurobehavioral mechanisms by which stress facilitates the reactivation of these aversive memories was essential for the development of targeted interventions aimed at preventing relapse in individuals with opioid dependence. As noted above, memories can contribute to relapse in drug addiction [4], but they do not directly address whether such modulation translates into reduced vulnerability to stress-induced opioid seeking or relapse-like behavior [33]. Our findings demonstrate that both psychosocial (social defeat) and physical stress paradigms (restraint and tail pinch) effectively reinstated conditioned place aversion (CPA) in morphine-dependent mice, indicating the reactivation of opioid withdrawal memory (Figure 2, Figure 3 and Figure 4). Importantly, pretreatment with the β-ARs antagonist propranolol attenuated this reinstatement (Figure 5), suggesting that β-ARs pathways play an important role in stress-induced memories that may contribute to relapse.

It is widely known that propranolol is a non-selective β-adrenergic receptor antagonist with well-characterized systemic effects that extend beyond memory modulation. Peripherally, blockade of β1-adrenergic receptors in the heart leads to reductions in heart rate, myocardial contractility, and cardiac output, as well as decreased blood pressure, reflecting its established clinical use in the treatment of hypertension, arrhythmias, and ischemic heart disease. β2-adrenergic blockade contributes to peripheral vasoconstriction and, in some contexts, can affect respiratory function by reducing bronchodilation. Centrally, propranolol readily crosses the blood–brain barrier due to its high lipophilicity, allowing it to influence arousal and stress-related processes mediated by central noradrenergic signaling [17]. By these effects, we suggest that it would attenuate stress-induced autonomic activation and modulate anxiety-like behavior.

Together with the brain stress system, they play a prominent role in regulating both systems [34]. In fact, the activity of the noradrenergic system is enhanced during chronic opioid dependence and during morphine withdrawal. Interestingly, the role of β-ARs in addiction and relapse has been less extensively studied than that of CRF; emerging evidence suggests their involvement in stress-induced drug-seeking behavior [11,35]. In our study, pretreatment with propranolol, a non-selective β-adrenergic antagonist, prior to stress exposure, attenuated the reinstatement of opioid withdrawal memory, as evidenced by the increased time spent in the naloxone-paired compartment during the reinstatement test (Figure 5B,D,F). This effect was observed across all three experimental groups chronically treated with morphine, which displayed naloxone-induced aversion when propranolol was administered prior to exposure to either physical or psychosocial stress. Notably, the three-way ANOVA revealed differential interaction patterns between chronic treatment, acute treatment, and stress exposure depending on stress modality. Specifically, no significant three-way interaction was detected under psychosocial stress conditions (social defeat), whereas significant main effects were identified for acute treatment and stress type. In contrast, no significant changes were seen among the different experimental groups not exposed to stress for this variable (Figure 5B,D,F). These findings, indicating an attenuation of the effects of physical and psychosocial stress, were consistent with our expectations based on previous studies using β-ARs antagonists. In contrast, slight but non-significant increases in this variable were observed in the social defeat–saline and restraint–saline groups exposed to stress when compared with their respective control groups (Figure 5B,D). We suggest that this effect may not be due to changes in locomotor activity, as inter-compartmental crossings were not altered (Figure 5A,C,E), but was most likely due to the blocking of conditioned memories. Nevertheless, this kind of measure does not exclude changes in overall activity levels, exploratory behavior, or arousal.

The interaction between β-adrenergic signaling and the CRF system has been widely documented. For instance, β-adrenergic activation can enhance CRF release in the brain, and CRF can, in turn, modulate β-adrenergic receptor expression and function [14]. This bidirectional interaction suggests that β-ARs may modulate stress-induced reinstatement of aversive memories that contribute to relapse through CRF-dependent mechanisms. Our findings contribute to this understanding by demonstrating that β-AR blockade can attenuate stress-induced reinstatement of opioid withdrawal memory, potentially through interactions with the CRF system, though likely not exclusively.

The antagonism of β-AR may modulate physiological and emotional responses to stress that facilitate memory reactivation. Our results are in line with previous studies from different laboratories that identify β-ARs as potential pharmacological targets to weaken maladaptive memories related to substance use disorders. Preclinical research has shown that administration of propranolol following memory reactivation significantly decreases the motivational value of drug-associated stimuli. For example, in rodent models, propranolol has been shown to suppress cocaine and heroin seeking by interfering with the reconsolidation of conditioned place preference and self-administration memories [22,36]. Another study revealed that propranolol affected both the consolidation and reconsolidation of aversive memories in mice but did not reliably reduce conditioned fear responses [37]. Furthermore, selective blockade of β1-ARs has been shown to disrupt cocaine-associated memory retrieval and retention [23]. Taherian et al. (2014) further validated the role of propranolol in modulating fear-related memory processes, demonstrating that its administration to rats disrupted the reconsolidation of contextual fear memories [38]. Importantly, the impairment was evident in both recently acquired and long-term memories, suggesting lasting therapeutic potential. In addition, propranolol administered following the reactivation of conditioned fear memory in Wistar rats led to a progressive attenuation of fear expression across repeated sessions [39]. Similarly, in clinical studies, propranolol has reduced context-induced craving in addicted subjects to nicotine [40] and also has attenuated alcohol-related memory reactivation. In contrast, propranolol has failed in another study to produce significant reductions in craving or alcohol-seeking behavior [41].

Our findings, together with those from other laboratories, support a role for propranolol in addiction-related memories, but several limitations advise further research. For instance, using a selective β1-AR and/or β2-AR antagonist is recommended to more precisely assess the contribution of these receptors to stress-induced relapse phenomena related to negative memories. Moreover, the duration and the level of morphine exposure, as well as the length of the abstinence period prior to stress induction, probably modulate vulnerability to relapse. Although aversive affective states and fear of withdrawal are widely recognized as important contributors to opioid relapse, the present findings should be interpreted within the specific constraints of the behavioral model employed. The conditioned place aversion (CPA) paradigm used here assesses the re-emergence of an aversive contextual memory associated with naloxone-precipitated morphine withdrawal. Furthermore, given that there are gender differences in stress response and addiction-related behaviors, this research cannot be generalized to females. Finally, although the present study is focused on the behavioral signs of stress-induced that influence the appearance of relapse, further research is needed to elucidate the functions of key brain regions, such as the prefrontal cortex, hippocampus, and amygdala, and the intracellular signaling cascades operating within these structures that are involved in relapse.

## 4. Materials and Methods

### 4.1. Animals

Adult male C57BL/6J mice (*n* = 179), which weighed 25–30 g at the beginning of the experiment, were obtained from the Animal Facilities of the University of Murcia (CEIB), Murcia, Spain. These animals were divided into groups of 4–6 per cage under controlled temperature conditions, with access ad libitum to food and water. A 12-h light/dark cycle was established throughout the study. Prior to the experimental procedures, mice were handled and exposed to the testing environment for at least one week. All procedures in this study have adhered to the European Communities Council Directive 2010/63/EU (22 September 2010) and were approved by the Animal Ethics Committee of the University of Murcia (CEEA 534/2019).

Mice were randomly assigned to one of two treatment conditions: chronic saline (*n* = 86) or chronic morphine (*n* = 93). With the purpose of inducing dependence on morphine, the drug was administered intraperitoneally once daily in escalating doses: 10 mg/kg on day 1, 30 mg/kg on day 2, 50 mg/kg on day 3, and 60 mg/kg on day 4. This dosage has been previously validated to induce opioid tolerance and dependence [30,42,43,44]. Instead, control animals received equivalent volumes of saline following the same schedule. Subsequently, animals were randomly assigned to acute treatment conditions (vehicle or propranolol) and according to stress type (physical, psychosocial, or control group). Behavioral scoring was performed blind to treatment, thereby strengthening the rigor of the experimental design.

### 4.2. Conditioned Place Aversion (CPA)

**Figure 6:**
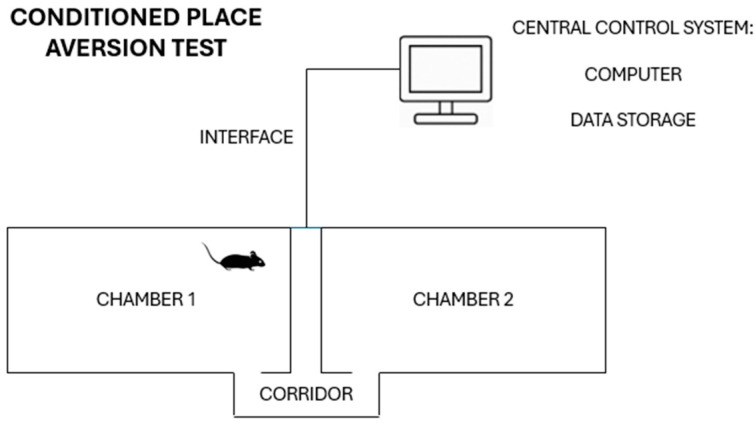
The figure shows a CPA equipment outline diagram.

**Figure 7:**
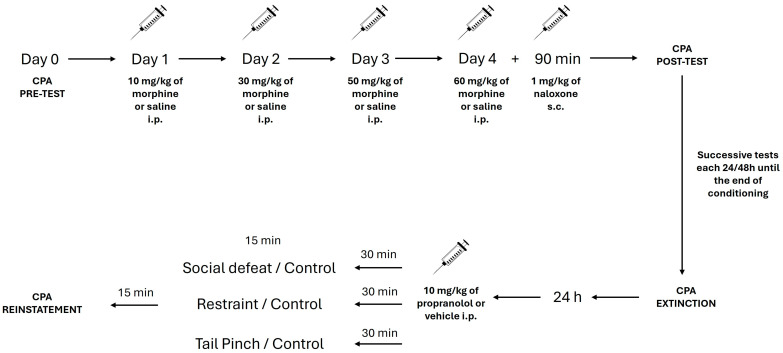
Protocol for determining the impact of social and physical stress on relapses in aversive memories related to opioid addiction by the performance of the CPA test: study of β-adrenergic receptors involvement (see Section 4).

The naloxone-induced CPA paradigm was used to assess aversive learning associated with opioid withdrawal. The CPA equipment consisted of two identical polyvinylcarbonate (PVC) boxes, subdivided into two conditioning chambers separated by a central corridor, and connected to a computerized tracking system (Figure 6) [45]. Distinct visual and tactile cues are displayed throughout the two main compartments: chamber 1 featured gray striped walls and a smooth black floor, while chamber 2 had black spotted walls and a rough gray floor. During all test sessions, animals were allowed unrestricted movement throughout the apparatus. The CPA protocol comprised five phases: pre-test, drug administration, conditioning, post-test, and extinction (Figure 7).

On day 0 (pre-test), mice were placed in the central corridor and allowed to freely explore the three compartments for 15 min. The time spent in each compartment was registered, as well as the number of crossings from one to another. Animals exhibiting a strong initial preference for either chamber (i.e., spending <360 s in one compartment) were excluded from subsequent analysis (*n* = 2).

From days 1 to 4, mice were injected with morphine or saline as described previously. On day 4, 90 min after the final morphine injection, naloxone (1 mg/kg, s.c.) was administered to precipitate withdrawal. The naloxone dose has been previously validated, demonstrating robust CPA induction in C57BL/6J mice [30,41]. Immediately following injection, animals were confined to one of the two conditioning chambers for 15 min.

CPA expression was evaluated on day 5 under drug-free conditions by using the same procedure as in the pre-test. The time spent in the naloxone-paired chamber was recorded and registered.

### 4.3. Extinction of CPA

Extinction sessions began 24 h after the CPA post-test and were conducted daily for 20–25 days until extinction criteria were met. Extinction was confirmed using paired t-test analyses comparing post-test and final extinction values. The procedure during each session started when the mice were placed in the central passageway and allowed free access to all chambers for 15 min. Time spent in the naloxone-paired chamber and the number of crossings between compartments were recorded.

### 4.4. Reinstatement of Conditioned Place Aversion

Reinstatement refers to the re-emergence of a previously extinguished conditioned response following exposure to an unconditioned stimulus—such as a drug, drug-associated cues, or stressors—when extinction has previously occurred [31,46]. In the present study, reinstatement of naloxone-induced CPA was evaluated one day after extinction by exposing mice to either an acute social defeat or a physical stressor (restraint or tail-pinch). The reinstatement test followed the same procedure as the pre-test, post-test, and extinction sessions, allowing animals free access to all compartments of the apparatus for 15 min. To minimize contextual associations, all reinstatement procedures were conducted in a room distinct from that used during conditioning and extinction phases, thereby ensuring a non-contingent spatial context relative to the original drug-paired environment.

### 4.5. Experimental Groups

#### 4.5.1. Experiment 1: Pharmacological Modulation of Reinstatement Through Administration of Propranolol

Only one day after extinction, mice were randomly assigned to receive either the β-ARs antagonist propranolol at a dose of 10 mg/kg (i.p.) or its vehicle (saline) (Figure 7). The selected dose was based on previous studies that have shown efficacy to reduce the stress-induced reinstatement of aversive memories [36,47,48]. Forty-five minutes after the administration, animals were subjected to the reinstatement test.

#### 4.5.2. Experiment 2: Role of β-ARs Signaling in Social Defeat-Induced Reinstatement

To assess the impact of social stress on the reinstatement of naloxone-induced CPA, mice were exposed to a 15-min agonistic encounter with an aggressor of similar age and weight (Figure 7). These aggressors were previously housed individually, screened for high levels of aggressive behavior, and had prior fighting experience. During the encounter, experimental mice displayed characteristic defensive and submissive behaviors, including avoidance or fleeing [49]. All defeated animals experienced comparable levels of aggression, with attacks that were initiated within 30 s of introduction. No abnormal behaviors were observed in control or aggressor animals.

As a control group for the potential nonspecific effects of social interaction, an additional group of mice was exposed to a non-aggressive social encounter with a familiar conspecific previously housed in a group [50]. These interactions, which lasted 15 min in a neutral transparent cage, served as a baseline for normal social interaction.

Aiming to investigate the involvement of β-ARs signaling in stress-induced reinstatement, a subset of mice received an acute injection of propranolol (10 mg/kg, i.p.) 30 min prior to the social defeat procedure. Control animals received vehicle injections. Fifteen minutes after exposure to psychosocial stress, all animals were subjected to the reinstatement test.

#### 4.5.3. Experiment 3: Impact of β-AR Antagonism on Restraint Stress-Induced Reinstatement of Naloxone-Paired Conditioned Place Aversion

One day after the extinction phase, mice received an acute intraperitoneal injection of either the non-selective β-ARs antagonist propranolol or its vehicle. Then, after thirty minutes (Figure 7), animals were subjected to a restraint stress protocol, which involved individual confinement within well-ventilated 50 mL polypropylene conical tubes for a duration of 15 min (physical stress). The restraint tube permitted adequate breathing while the head and limb movement were restricted. When the stress exposure ended, the animals returned to their home cages. A control group remained undisturbed in their home cages for the same time interval. Finally, the reinstatement test was displayed for all the animals.

#### 4.5.4. Experiment 4: Effect of β-AR Blockade on Tail-Pinch Stress-Induced Reinstatement of Naloxone-Associated CPA

Twenty-four hours after extinction, mice were acutely administered propranolol or its vehicle. After thirty minutes (Figure 7), physical stress was induced via tail-pinch, by affixing a 3.2-cm paper clip approximately 2 cm in length from the distal end of the tail for a period of 15 min. A non-stressed control group remained in their home cages during the same time frame. After stress exposure, all animals were evaluated in the reinstatement test (Figure 7).

### 4.6. Statistical Analysis

All data are expressed as mean ± standard error of the mean (SEM). Group comparisons were conducted using one-way analysis of variance (1-way ANOVA) or three-way analysis of variance (3-way ANOVA), followed by Tukey post hoc testing when it was appropriate. For comparisons involving only two groups, Student’s *t*-test was performed. Statistical significance was established as *p* < 0.05.

## 5. Conclusions

The present findings highlight an important role for β-ARs in the modulation of the stress-induced reinstatement of opioid withdrawal memory, which may affect relapse vulnerability following prolonged periods of abstinence. Hence, targeting these receptors may offer novel therapeutic options for preventing memories that contribute to relapses in opioid use disorder. Further research is needed to elucidate the neural mechanisms involved, but also to confirm the clinical efficacy of β-ARs antagonists.

## Figures and Tables

**Table 1:** Behavioral profiles after morphine withdrawal precipitated by naloxone (Nx).

Withdrawal Signs	Saline + Nx	Morphine + Nx
Rinorrhea	0/86	88/93 ***
Spontaneous jumping	0/86	86/93 ***
Salivation	0/86	86/93 ***
Ptosis	0/86	90/93 ***
Diarrhea	0/86	84/93 ***
Wet-dog shakes	0/86	85/93 ***
Teeth-chattering	0/86	91/93 ***
Piloerection	0/86	89/93 ***
Tremor	0/86	92/93 ***
Chromodacryorrhea	0/86	79/93 ***

*** *p* < 0.001 vs. Saline + Nx.

**Table 2:** Detailed statistics for Figure 5 data.

Group	C-SD	*p*	SD	*p*	C-R	*p*	R	*p*	C-TP	*p*	TP	*p*
Sal + Nx + Veh	*n* = 5 ^a^	n.s.	*n* = 7 ^e^	n.s.	*n* = 8 ^i^	n.s.	*n* = 6 ^m^	n.s.	*n* = 7 ^q^	n.s.	*n* = 7 ^u^	n.s.
Sal + Nx + Prop	*n* = 7 ^b^	n.s.	*n* = 8 ^f^	n.s.	*n* = 8 ^j^	n.s.	*n* = 8 ^n^	n.s.	*n* = 7 ^r^	n.s.	*n* = 8 ^v^	n.s.
Mor + Nx + Veh	*n* = 5 ^c^	n.s.	*n* = 6 ^g^	0.0305 vs. c	*n* = 10 ^k^	n.s.	*n* = 9 ^o^	0.0000 vs. k 0.0207 vs. m	*n* = 9 ^s^	n.s.	*n* = 8 ^w^	0.0255 vs. s
Mor + Nx + Prop	*n* = 8 ^d^	n.s.	*n* = 8 ^h^	0.0105 vs. g	*n* = 8 ^l^	n.s.	*n* = 6 ^p^	0.0035 vs. o	*n* = 8 ^t^	n.s.	*n* = 8 ^x^	0.0085 vs. w
TOTAL	*n* = 25		*n* = 29		*n* = 34		*n* = 29		*n* = 31		*n* = 31	

*p* (*p*-value for Tukey’s analysis test); C-SD (control group for Social Defeat); SD (Social Defeat group); C-R (control group for Restraint); R (Restraint Group); C-TP (control group for Tail Pinch); TP (Tail Pinch); a–x (letter of identification for every experimental group).

## Data Availability

The original contributions presented in this study are included in the article. Further inquiries can be directed to the corresponding author.

## Abbreviations

The following abbreviations are used in this manuscript:

β-AR	Beta-Adrenergic Receptor
CPA	Conditioned Place Aversion
CPP	Conditioned Place Preference
CRF	Corticotropin-Releasing Factor
